# Pain and Interventions in Stage IV Non-Small Cell Lung Cancer: A Province-Wide Analysis

**DOI:** 10.3390/curroncol30030262

**Published:** 2023-03-18

**Authors:** Vivian S. Tan, Michael C. Tjong, Wing C. Chan, Michael Yan, Victoria Delibasic, Gail Darling, Laura E. Davis, Mark Doherty, Julie Hallet, Biniam Kidane, Alyson Mahar, Nicole Mittmann, Ambika Parmar, Hendrick Tan, Frances C. Wright, Natalie G. Coburn, Alexander V. Louie

**Affiliations:** 1 Department of Radiation Oncology, University of Western Ontario, London, ON N6A 5W9, Canada; 2 Department of Radiation Oncology, Dana-Farber Cancer Institute and Brigham and Women’s Hospital, Boston, MA 02215, USA; 3 ICES, Toronto, ON M4N 3M5, Canada; 4 Department of Radiation Oncology, University of Toronto, Toronto, ON M5G 1P5, Canada; 5 Division of Thoracic Surgery, University of Toronto, Toronto, ON M5G 1P5, Canada; 6 Department of Epidemiology, Biostatistics and Occupational Health, McGill University, Montreal, QC H3A 1G1, Canada; 7 Department of Medical Oncology, University of Toronto, Toronto, ON M5S 3H2, Canada; 8 Department of Surgery, University of Toronto, Toronto, ON M5G 1P5, Canada; 9 Division of Thoracic Surgery, University of Manitoba, Winnipeg, MB R3A 1R9, Canada; 10 Faculty of Health Sciences, Queen’s University, Kingston, ON K7L 3N6, Canada; 11 Canadian Agency for Drugs and Technology in Health, Ottawa, ON K1S 5S8, Canada; 12 Department of Radiation Oncology, GenesisCare, Perth, WA 6000, Australia

**Keywords:** non-small cell lung cancer, pain, quality of life, patient-reported outcomes

## Abstract

Pain is a common symptom in stage IV non-small cell lung cancer (NSCLC). The objective of the study was to examine the use of interventions and factors associated with interventions for pain. A population-based cohort study in Ontario, Canada was conducted with patients diagnosed with stage IV NSCLC from January 2007 to September 2018. An Edmonton Symptom Assessment System (ESAS) score of ≥4 defined moderate-to-severe pain following diagnosis. The study cohort included 13,159 patients, of which 68.5% reported at least one moderate-to-severe pain score. Most patients were assessed by a palliative care team (85.4%), and the majority received radiation therapy (73.2%). The use of nerve block was rare (0.8%). For patients ≥65 years of age who had drug coverage, 59.6% received an opiate prescription. Patients with moderate-to-severe pain were more likely to receive palliative assessment or radiation therapy compared to patients with none or mild pain. Patients aged ≥70 years and with a greater comorbidity burden were associated with less likelihood to receive radiation therapy. Patients from rural/non-major urban residence and with a greater comorbidity burden were also less likely to receive palliative care assessment. Factors associated with interventions for pain are described to inform future symptom management in this population.

## 1. Introduction

Lung cancer leads in cancer incidence and mortality worldwide [1]. Nearly half of non-small cell lung cancer (NSCLC) presents as stage IV [2], which has historical estimates of 15% of patients surviving 5 years from diagnosis [3]. In addition to poor prognosis, lung cancer is associated with a high symptom burden. Pain is a common symptom that significantly impacts a patient’s quality of life, with an overall weighted mean prevalence of pain of 47% in all lung cancer patients [4]. In addition, a previous study in Ontario described high rates of hospital admissions and emergency room visits in lung cancer patients nearing end-of-life and pain as one of the most common chief complaints [5]. Therefore, symptom management is a priority in the management of lung cancer.

Palliative care is commonly involved to help providing pain management strategies in cancer patients. First-line treatment for cancer pain often includes analgesics, such as opioids. If pain is related to bony metastasis, palliative radiotherapy may be considered. For patients who do not experience relief with first-line treatments, patients may be referred to interventional anesthesia for consideration of nerve blocks [6].

Patient-reported outcomes (PROs) describe the burden of symptoms from the patient’s perspective. Routine documentation of PROs has been associated with early identification of symptoms, improved quality of life and improved survival outcomes [7,8,9,10]. PROs are also associated with decreased emergency department visits in patients with advanced cancers [11]. The Edmonton Symptom Assessment System (ESAS) is a validated patient-reported outcome tool that assesses severity on a scale from 0 to 10 of nine common cancer symptoms. These symptoms include anxiety, depression, drowsiness, lack of appetite, nausea, pain, shortness of breath, tiredness and impaired well-being. ESAS questionnaires have been collected at outpatient clinic visits in Ontario, Canada since 2007 [12].

Overall symptom burden using ESAS in stage IV NSCLC in Ontario has been previously reported by our research group, which found moderate-to-severe symptoms were prevalent and peaked at diagnosis while remaining high during the first year of follow-up [13]. The aim of the current study was to examine the intervention use and factors associated with interventions for pain (including palliative care assessment, radiation therapy, nerve block and opiate prescriptions) in stage IV NSCLC. This will allow us to identify populations that are less likely to receive pain treatment and target improvements in the cancer system to address these gaps.

## 2. Materials and Methods

### 2.1. Study Design

Routinely collected data held at ICES (formally the Institute for Clinical Evaluative Sciences) in Ontario, Canada were used to conduct this population-based cohort study. The study was conducted according to approval by the Research Ethics Board at Sunnybrook Health Sciences Centre (Toronto, ON, Canada).

### 2.2. Data Sources

Patients with NSCLC were identified using the Ontario Cancer Registry (OCR). ESAS scores were obtained from the Cancer Care Ontario Symptom Management Reporting Database that has systematically collected questionnaires from outpatient cancer clinic visits at regional cancer centers beginning in 2007. Demographic data were retrieved from the Ontario Registered Persons Database (RPDB) and the Canadian Census. Billing claims for physician services were obtained from the Ontario Health Insurance Plan (OHIP) database. 

The Cancer Activity Level Reporting (ALR) was used to capture information regarding radiation treatments. Information from hospitalizations was retrieved from the Canadian Institute for Health Information Discharge Abstract Database (DAD), while emergency room visits were retrieved from the National Ambulatory Care Reporting System (NACRS). Outpatient prescriptions dispensed to individuals aged ≥65 years are captured in the Ontario Drug Benefit (ODB) database with an error rate of <1% [14], and this information was used to identify prescription drug use. Further information regarding the data sources and codes used in the study can be found in the Supplementary Materials.

### 2.3. Study Cohort

Our study cohort included patients diagnosed with stage IV NSCLC from January 2007 to September 2018 using the OCR. Exclusion criteria included age less than 18 years, no ESAS scores were collected following diagnosis or if the patient had another cancer diagnosis 5 years prior to diagnosis or during follow-up. Patients were followed from the date of diagnosis to date of last contact, death or end of the study timeframe (30 September 2019).

### 2.4. Baseline Characteristics

Baseline characteristics were measured at the time of diagnosis, including age, sex, rural residence, neighbourhood income quintile and comorbidity burden. Other factors included the year of diagnosis and number of ESAS questionnaires. Systemic therapy was defined as patients who had more than cycle of chemotherapy/immunotherapy. Death was defined by any cause recorded in the RPDB and administrative datasets, such as DAD and NACRS, during the follow-up period. The Rural Index of Ontario was used to measure rural residence. 

This index considers population size, population density and healthcare resources to stratify data into the following categories: major urban (0–9), non-major urban (10–44) and rural (45–100) [15]. Neighbourhood income quintiles were defined by the median income of the neighbourhood of the patient’s postal code at the time of diagnosis based on Census data. The Elixhauser Comorbidity Index was used to assess the medical comorbidities of patients based on the use of healthcare services within 24 months prior to the lung cancer diagnosis. The index is scored where a value ≥4 indicates a moderate-to-severe burden of comorbidities [16,17].

### 2.5. Variables and Outcomes

Moderate-to-severe pain was defined by an ESAS score ≥4, which has been previously validated to identify patients with significant symptoms [18]. Patients were considered to have moderate-to-severe pain if they had at least one ESAS score ≥4 from the date of diagnosis to the end of follow-up. Otherwise, they were considered to have none or mild pain. The outcomes of interest were interventions for pain, including palliative care assessment, radiation therapy, nerve block and opiate prescriptions in the study. 

Palliative care was defined by evidence of an assessment from OHIP, DAD or NACRS. Palliative care was further stratified by inpatient, outpatient and both categories. Radiation therapy and nerve block were identified from ALR and OHIP, respectively. These interventions were measured by a diagnostic code at any time from diagnosis to end of follow-up. The use of opiates was evaluated in patients ≥65 years of age who had coverage for prescription drugs through the ODB program. This included the dispensing of codeine, fentanyl, hydromorphone, morphine, oxycodone and tramadol.

### 2.6. Statistical Analysis

Baseline characteristics were presented for continuous variables in medians/interquartile ranges (IQR) and for categorical variables in counts/percentages. These characteristics were further stratified by patients who reported moderate-to-severe and none or mild pain scores and were then compared using standardized differences. A standardized difference >10% was considered as a meaningful difference. We also calculated the proportion of patients who underwent interventions, including palliative care assessment, radiation therapy, nerve block and use of opiates, for patients with moderate-to-severe pain scores and for patients with none or mild pain scores. Intervention use was also compared using standardized differences. 

Factors associated with the different intervention use among patients with moderate-to-severe pain were explored using multivariable modified Poisson regression models with robust error variance estimator for rates of the outcomes; the use of a Poisson model is a good approximation to binomial distribution without the convergence issues that are associated with negative binomial models. The relevant covariates controlled for in the model included age, sex, rural residence, neighborhood income quintile, comorbidity burden and year of diagnosis. These variables included in the model were determined a priori based on clinical relevance. The results are reported as relative risks (RR) and 95% confidence intervals. All analyses were conducted using SAS, version 9.4 (SAS Institute, Cary, NC, USA).

## 3. Results

### 3.1. Baseline Characteristics

**Figure 1:**
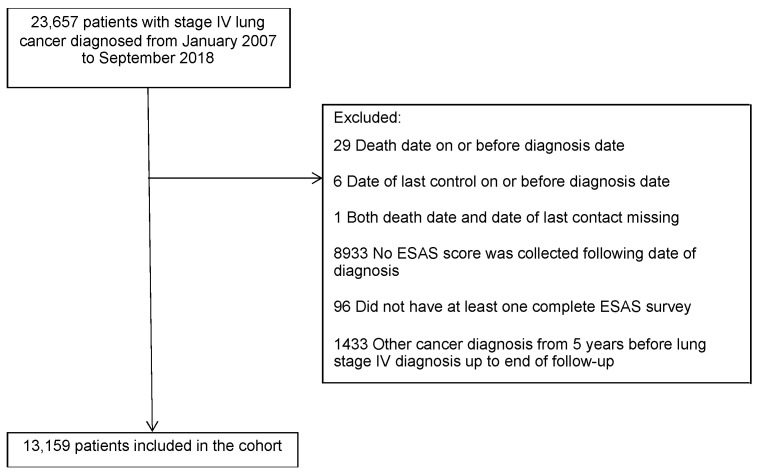
Study cohort.

In total, 13,159 patients diagnosed with stage IV NSCLC from January 2007 to September 2018 met our inclusion criteria (Figure 1). The median number of ESAS surveys completed was 4 (IQR: 2–11), and the median follow-up was 9 months (IQR: 4–18). In the cohort, 11,907 (90.5%) died in the study period.

The baseline characteristics stratified by ESAS pain score are summarized in Table 1. The reporting of at least one moderate-to-severe ESAS pain score was common (68.5%, *n* = 9008). Patients with moderate-to-severe pain scores were more likely to be younger (median age 67 vs. 70 years; standardized difference (SD) 30%) and completed ESAS questionnaires more frequently (six vs. two questionnaires; SD 60%). Patients with moderate-to-severe pain also more commonly received systemic therapy after diagnosis (63.1% vs. 52.2%; SD 22%). Other baseline characteristics, including sex, rural residence, income quintile and comorbidity burden, were not significantly different for patients who reported moderate-to-severe pain scores as compared to patients who did not report moderate-to-severe pain scores.

The median time from diagnosis to first moderate-to-severe pain score was 50 (IQR: 25–134) days, and the median time from first moderate-to-severe pain score to death was 131 (IQR: 55–291) days. Death was not significantly different in patients with moderate-to-severe pain scores compared to patients with none or mild pain (91.1% vs. 89.2%; SD 6%).

### 3.2. Interventions for Pain

**Figure 2:**
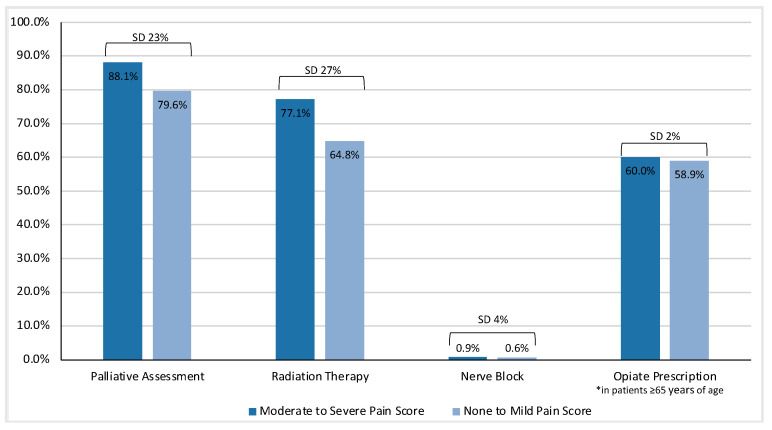
Intervention use stratified by patient-reported pain score. Standardized difference (SD).

Interventions based on ESAS pain scores are summarized in Table 2. In summary, 94.3% of the entire cohort received interventions for pain, which included palliative care assessment, radiation therapy and/or nerve block. Palliative care assessment (85.4%) and radiation therapy (73.2%) were common. Few patients received nerve blocks (0.8%). Patients with moderate-to-severe pain scores were significantly more likely to receive a palliative assessment (88.1% vs. 79.6%; SD 23%) or radiation therapy (77.1% vs. 64.8%; SD 27%) as demonstrated in Figure 2. Nerve block was not significantly different between patients with moderate-to-severe pain and those with none or mild pain scores (0.9% vs. 0.6%; SD 4%). Of patients who reported moderate-to-severe pain scores, 4.3% of patients did not receive any intervention for pain.

In an analysis of opioid use, which was restricted to 8061 patients ≥65 years of age who had prescription coverage through the ODB program, 59.6% received an opiate prescription. There was no significant difference in opiate prescriptions in patients with moderate-to-severe vs. none or mild pain scores (60.0% vs. 58.9%; SD 2%).

For those patients who received palliative care assessment, further stratification revealed that 2.4% received inpatient palliative care, 58.9% received outpatient palliative care, and 38.7% received both inpatient and outpatient palliative care. When characterizing by the type of palliative care that was received at the first setting, 17.6% of patients received inpatient first, while 82.4% received outpatient first. Palliative care stratified by type for patients with moderate-to-severe pain vs. none or mild pain is also reported in Table 2.

### 3.3. Factors Associated with Interventions for Pain

The results from the multivariable analysis to identify factors to receiving pain interventions are reported in Table 3. Compared to younger patients (age 18–59 years), older patients who reported moderate-to-severe pain were less likely to receive radiation therapy (age 70–79 years: RR 0.90 (0.88–0.93), age ≥80 years: RR 0.86 (0.82–0.90)). Nerve block use was 1.78 (1.14–2.79) times higher among female compared to male patients reporting moderate-to-severe pain. 

Greater comorbidity burden was associated with less likelihood to receive palliative care (RR 0.96 (0.93–1.0)) and radiation therapy (RR 0.90 (0.85–0.96)) in patients with moderate-to-severe pain. In addition, patients with moderate-to-severe pain from a rural (RR 0.86 (0.83–0.90)) or non-major urban (RR 0.95 (0.94–0.97)) residence were also less likely to receive palliative care. Overall, the association between neighborhood income quintile and intervention use was not significant. The use of palliative assessment increased (RR 1.003 (1.000–1.005)), while the use of radiation therapy decreased with year of diagnosis (RR 0.970 (0.966–0.974)).

## 4. Discussion

In this population-based study based in Ontario, Canada, 68.5% of stage IV NSCLC patients reported at least one moderate-to-severe ESAS pain score. Although the administrative data analyzed did not have sufficiently granular information on the etiology of pain, our findings are consistent with previously reported findings [19,20,21,22,23,24]. A systematic review and meta-analysis reported a prevalence of 66.4% of pain in advanced, metastatic or terminal disease in all cancer types [19].

Bubis et al. reported on the symptom burden in the first year of cancer diagnosis with a similar methodology in our healthcare system and found that 51% of respiratory cancers reported a pain ESAS score ≥4. The pain prevalence was higher than various other cancers compared, including GI, breast, gynecologic, genitourinary and CNS [20].

When compared in the metastatic context, NSCLC reported a higher prevalence of patients who reported moderate-to-severe ESAS pain scores compared with esophageal and gastric cancers [21,22]. Similarly, a retrospective cohort study that investigated the symptom burden in cancer patients in the last six months of life found that lung cancer had a higher risk of reporting severe pain (ESAS ≥ 7) when compared to colorectal, brain/central nervous system, stomach and esophagus cancers [23]. The pain burden in metastatic NSCLC was comparable to end-stage non-resected pancreatic cancer, which had a proportion of 67.3% of patients who reported moderate-to-severe ESAS pain scores in Ontario [24].

Our analysis showed that patients with moderate-to-severe pain were more likely to have a younger median age. Previous studies have also shown that older lung cancer patients were less likely to report pain, which was consistent with our results [25,26,27]. For example, Hirpara et al. found that patients over 80 years of age were significantly less likely to report severe pain than those under 50 years of age in non-metastatic lung cancer (RR 0.52 (0.42–0.65)) [28]. There are multiple possible factors that influence this finding, including that older patients may be less likely to express pain or that healthcare professionals are less likely to record this symptom in this age group [4]. Consistent with prior publications, sex was not associated with pain severity [27,29].

Most patients in our cohort were managed with palliative care (85.4%). Quinn et al. reported that the delivery of palliative care among cancer patients in their last year of life was delivered in the following settings: 1.5% inpatient, 6.9% outpatient, 3.4% home-based, 4.9% case management and 83.3% multiple locations. A total of 15.3% of patients received their first palliative care in setting of a hospital, which was similar to our reported inpatient rate of 17.6% [30]. Early palliative care has been associated with a survival benefit among patients with advanced lung cancer [31]. Temel et al. demonstrated, in a randomized controlled trial, that early palliative care improved symptom management and survival in metastatic NSCLC [32]. Early palliative care was also shown to have reduced acute hospital use and healthcare costs during end of life in our healthcare system [33,34].

ESAS has the potential role to identify patients early for palliative care. A retrospective-matched cohort study in Ontario found ESAS assessments were associated with a 6% increase in palliative care services in cancer patients [35]. Clinicians can use ESAS symptom assessments as a tool to trigger palliative care assessments. Goldie et al. conducted a retrospective cohort study of patients who died of NSCLC in Ontario and demonstrated that, although the number of patients receiving supportive care increased over the study period (2009–2017), 56% of the cohort did not receive palliative care consultation before death, and benchmarks for end-of-life quality indicators were not met [36].

Our study also demonstrated that patients with greater comorbidity or who were not from a major urban residence with moderate-to-severe pain were less likely to receive palliative care. There is variation of palliative care services across the province of Ontario. This relationship was previously reported in a study by Tanuseputro et al. who found that patients living in rural regions in Ontario were less likely to receive palliative care (OR 0.80 (0.78–0.83)) [37]. In a study by Conlon et al. that looked at access to palliative care for cancer patients in rural Ontario, they also found that northern rural residents were less likely to receive palliative care [38]. Factors that may influence this finding include geography, workforce and access to cancer care resources. This supports the continued importance of increasing resources to improve access to palliative care in rural locations.

Radiation therapy was a common therapeutic intervention for pain in our cohort (73.2%). Our findings are consistent with the utilization rates of palliative radiation among patients with metastatic NSCLC in the United States, which have been reported to be 50% or greater [39,40]. Older patients or patients with greater comorbidity who reported moderate-to-severe pain were less likely to receive radiation therapy. In the literature, older patients were less likely to receive radiation for curative treatment in other stages of lung cancer as well [41]. 

The negative association between age and palliative radiation was also reported in a previous study in Ontario amongst all cancers [42]. However, patients should not be precluded from receiving radiotherapy based on age and comorbidities alone. Palliative radiotherapy is a well-tolerated treatment for symptom control that can be beneficial even for patients with poor performance status. In addition, there is growing evidence that the use of radiation in oligometastatic disease is associated with increased overall survival [43].

Symptom management is important in the metastatic lung cancer population considering the high proportion of patients diagnosed at this stage and the associated poor prognosis. In this study, 4.3% of patients who reported moderate-to-severe pain did not receive any intervention for pain highlighting the unmet need in this population. With the shift to virtual care since the COVID-19 pandemic, there is an opportunity to utilize patient-reported outcomes to improve care for patients. This includes the virtual collection and monitoring of ESAS scores. During the pandemic, virtual collection of ESAS though emailing a fillable PDF form was reported in a prospective study at Princess Margaret Hospital in Toronto, Ontario [44]. Other possible methods to collect ESAS from home could include phone assessments, web-based forms and virtual applications.

High symptom scores can be used to trigger automatic assessments of patients. The Lurie Cancer Center Gynecologic Oncology outpatient clinic linked PRO assessments to the electronic healthcare record. Patients completed computer adaptive tests to assess symptoms, and clinicians were notified of elevated scores and triaged to social work, health educators and dieticians depending on the concern [45]. A similar clinical workflow can be implemented in cancer centers in Ontario, particularly where severe ESAS pain scores can automatically trigger physician/nursing assessments and referrals to palliative care. 

ESAS can also be used to triage patients for home-based palliative care following a referral. Dhiliwal et al. completed a pilot of a triage coding system where high priority (ESAS ≥ 7) were seen within 3 days, medium priority (ESAS 4–6) were seen within 10 days and low priority (ESAS 0–3) were seen within 15 days [46]. This has the potential to identify and intervene patients in the community earlier and, thus, prevent acute care services, such as emergency room visits and hospital admissions, in this population for pain.

Our study has several strengths. First, we used data from a province-wide implementation of ESAS patient-reported outcomes to routine clinical care, allowing for a large sample size. Second, in the setting of universal healthcare coverage, our study diminished cofounders, such as direct financial barriers preventing patients receiving interventions for pain. Limitations of our study are also recognized. First, a large proportion of patients were excluded as ESAS scores were not collected. As described previously, patients who did not complete any ESAS were more likely to be older, to have higher comorbidity and to not receive any active cancer treatment [13]. 

Therefore, we may be underestimating the true pain burden in the population. Second, our findings regarding nerve block were limited by the small sample size of patients who underwent this intervention in our study cohort. Third, we were restricted by administrative data. For example, we only had data available to describe opiate prescription in the ≥65 years age cohort. 

As a result, we are unable to accurately characterize opioid prescription in younger adults diagnosed with stage IV lung cancer. Additional variables that could have helped better define the cohort (such as ethnicity or biomarkers) or describe other cancer interventions were not available. In addition, location descriptions of patient pain were not available. Lastly, we analyzed the symptom burden using the ESAS score at one time point. As a result, we did not follow pain across the course of disease progression and treatment.

## 5. Conclusions

A majority of patients with stage IV NSCLC reported moderate-to-severe pain (68.5%). Most patients received palliative care assessment or radiation therapy, while nerve block was rare. Patients from rural or non-major urban residences and patients with more comorbidities were less likely to receive palliative care assessment, while patients with older age and more comorbidities were less likely to receive radiation therapy. The data presented in this study help to identify gaps in our cancer care and suggest a continued need for pathways to ensure equitable supports for patients with a high pain burden.

## Figures and Tables

**Table 1:** Baseline characteristics for patients with and without moderate-to-severe pain ESAS scores.

Characteristics	None or Mild Pain ESAS Score (*n* = 4151)	Moderate-to-Severe Pain ESAS Score (*n* = 9008)	Standardized Difference
Sex			
Female	2038 (49.1%)	4388 (48.7%)	0.01
Male	2113 (50.9%)	4620 (51.3%)	0.01
Age	70 (63–78)	67 (59–74)	0.30
Residence			
Major urban	2634 (63.5%)	5986 (66.5%)	0.06
Non-major urban	1156 (27.8%)	2362 (26.2%)	0.04
Rural	345 (8.3%)	629 (7.0%)	0.05
Neighborhood Income Quintile			
Q1	849 (20.5%)	1968 (21.8%)	0.03
Q2	942 (22.7%)	2019 (22.4%)	0.01
Q3	829 (20.0%)	1744 (19.4%)	0.02
Q4	757 (18.2%)	1694 (18.8%)	0.01
Q5 (highest income)	759 (18.3%)	1558 (17.3%)	0.03
Elixhauser Comorbidity Index			
4 or more	3871 (93.3%)	8450 (93.8%)	0.02
Less than 4	280 (6.7%)	558 (6.2%)	0.02
Year of Diagnosis			
2007 to 2012	1652 (39.8%)	3502 (38.9%)	0.02
2013 to 2018	2499 (60.2%)	5506 (61.1%)	0.02
Number of ESAS questionnaires	2 (1–6)	6 (2–14)	0.60
Systemic Therapy	2165 (52.2%)	5686 (63.1%)	0.22
Death	3703 (89.2%)	8204 (91.1%)	0.06

Data are presented as *n* (%) or median (IQR). Standardized difference >10% considered to be a meaningful difference.

**Table 2:** Intervention use for patients with and without moderate-to-severe pain ESAS scores.

Interventions	Total Cohort	None or Mild Pain ESAS Score	Moderate-to-Severe Pain ESAS Score	Standardized Difference
Intervention for Pain	*n* = 13,159	*n* = 4151	*n* = 9008	
Yes	12,410 (94.3%)	3785 (91.2%)	8625 (95.7%)	0.19
No	749 (5.7%)	366 (8.8%)	383 (4.3%)	
Palliative Care				0.23 0.05 0.06 0.07
Assessment				
Yes	11,239 (85.4%)	3303 (79.6%)	7936 (88.1%)	
Inpatient	275 (2.4%)	101 (3.1%)	174 (2.2%)	
Outpatient	6616 (58.9%)	2008 (60.8%)	4608 (58.1%)	
Both	4348 (38.7%)	1194 (36.1%)	3154 (39.7%)	
No	1920 (14.6%)	848 (20.4%)	1072 (11.9%)	
Radiation Therapy				
Yes	9635 (73.2%)	2691 (64.8%)	6944 (77.1%)	0.27
No	3524 (26.8%)	1460 (35.2%)	2064 (22.9%)	
Nerve Block				
Yes	106 (0.8%)	24 (0.6%)	82 (0.9%)	0.04
No	13,053 (99.2%)	4127 (99.4%)	8926 (99.1%)	
For patients ≥65		*n* = 2863	*n* = 5198	0.02
Opiate Prescription				
Yes	4806 (59.6%)	1685 (58.9%)	3121 (60.0%)	
No	3255 (40.4%)	1178 (41.1%)	2077 (40.0%)	

Data are presented as *n* (%). Standardized difference >10% considered to be a meaningful difference. Standardized difference compared between none or mild pain ESAS score and moderate-to-severe pain ESAS score.

**Table 3:** Multivariable modified Poisson model for patients who reported moderate-to-severe pain ESAS scores.

Characteristics	Palliative Assessment	Radiation Therapy	Nerve Block	Use of Opiates (65+)
Sex				
Male	1.0 (reference)	1.0 (reference)	1.0 (reference)	1.0 (reference)
Female	1.006 (0.991–1.022)	0.991 (0.969–1.013)	1.782 (1.139–2.787)	1.014 (0.969–1.060)
Age (years)				
18–59	1.0 (reference)	1.0 (reference)	1.0 (reference)	1.0 (reference)
60–69	0.997 (0.977–1.017)	0.976 (0.951–1.001)	1.009 (0.586–1.735)	1.0 (reference)
70–79	1.000 (0.98–1.021)	0.902 (0.875–0.929)	0.586 (0.309–1.111)	0.980 (0.932–1.032)
80 and older	1.001 (0.974–1.028)	0.861 (0.822–0.901)	1.005 (0.488–2.068)	0.989 (0.926–1.056)
Residence				
Major urban	1.0 (reference)	1.0 (reference)	1.0 (reference)	1.0 (reference)
Non-major urban	0.953 (0.935–0.97)	1.006 (0.981–1.032)	1.052 (0.657–1.686)	0.972 (0.922–1.024)
Rural	0.864 (0.828–0.902)	1.015 (0.972–1.061)	0.177 (0.025–1.273)	0.995 (0.91–1.088)
Neighborhood Income Quintile				
Q1	0.978 (0.955–1.001)	0.972 (0.937–1.007)	0.578 (0.292–1.144)	0.961 (0.895–1.031)
Q2	0.973 (0.950–0.997)	0.98 (0.946–1.015)	0.638 (0.334–1.221)	0.959 (0.895–1.029)
Q3	0.992 (0.968–1.016)	0.982 (0.947–1.018)	0.885 (0.475–1.649)	0.957 (0.891–1.028)
Q4	0.987 (0.963–1.011)	1.014 (0.980–1.050)	0.552 (0.271–1.125)	0.969 (0.901–1.042)
Q5 (highest)	1.0 (reference)	1.0 (reference)	1.0 (reference)	1.0 (reference)
Elixhauser Cormorbidity Index				
Less than 4	1.0 (reference)	1.0 (reference)	1.0 (reference)	1.0 (reference)
4 or more	0.96 (0.926–0.996)	0.903 (0.853–0.957)	1.354 (0.595–3.083)	0.921 (0.844–1.006)
Year of Diagnosis				
2007 to 2018	1.003 (1.000–1.005)	0.970 (0.966–0.974)	0.977 (0.909–1.049)	0.998 (0.991–1.006)

Data are presented as RR (95% CI).

## Data Availability

The dataset from this study is held securely in coded form at ICES. While legal data sharing agreements between ICES and data providers (e.g., healthcare organizations and the government) prohibit ICES from making the dataset publicly available, access may be granted to those who meet pre-specified criteria for confidential access, available at www.ices.on.ca/DAS (accessed on 10 January 2023) (email: das@ices.on.ca). The full dataset creation plan and underlying analytic code are available from the authors upon request, understanding that the computer programs may rely upon coding templates or macros that are unique to ICES and are therefore either inaccessible or may require modification.

## Supplementary Materials

The following supporting information can be downloaded at: https://www.mdpi.com/article/10.3390/curroncol30030262/s1, Table S1: Database codes in Ontario healthcare databases.
