# Supplementary material for: Pain and Interventions in Stage IV Non-Small Cell Lung Cancer: A Province-Wide Analysis
Source: Curr Oncol. 2023 Mar 18;30(3):3461–72. doi: 10.3390/curroncol30030262 (PMC10047317; doi:10.3390/curroncol30030262)
Supplement: Supplementary file 1 [file curroncol-30-00262-s001.zip › curroncol-2186405-supplementary.pdf]

Supplementary Material

Table S1. Database codes in Ontario healthcare databases.

Characteristic	Database	Codes
Lung Cancer	Ontario Cancer Registry (OCR)	ICD-O-3 topography:<br>C34.0, C34.1, C34.2, C34.3, C34.8, C34.9<br>ICD-O-3 histology:<br>Adenocarcinoma:<br>81403, 81406, 83103, 84803, 84813, 82433, 84803, 82503, 82513, 82523, 82533, 82543<br>Squamous Cell Carcinoma:<br>80703, 80706, 80713, 80723, 80733, 80843, 80753, 80833<br>Large Cell Carcinoma:<br>80123, 80133, 80143, 82463<br>Adenosquamous Carcinoma:<br>85603
Palliative Care	CIHI-Discharge Abstract Database (DAD)	00121, 58
	National Ambulatory Care Reporting System (NACRS)	S00121
	Ontario Health Insurance Plan (OHIP)	A945, B966, B997, B998, C945, C982, G511, K015, K023, W982
Radiation Therapy	Cancer Activity Level Reporting (ALR)	National Health Productivity Improvement Program:<br>519, 530, 531, 532, 533, 534, 535, 536, 537, 538, 539, 540, 541, 542, 547, 548, 549, 575, 592, 594, 596, 597
Nerve Block	OHIP	G910, G911, G912, G913, G914, G915, N556, G917, G246, G117, G233, G279, G231
Opiates	Ontario Drug Benefit (ODB)	Drug Information Number:<br>Codeine:<br>00293504, 00293512, 00653241, 00653276, 02163934, 02163926, 00816027, 02163942, 00666130, 00608882, 00621463, 02163918, 00176206, 00176192, 00608181, 00608203, 02239645, 00779458, 00593435, 00593451, 02230302, 02163748, 02163780, 02163799<br>Fentanyl:<br>02314630, 02314649, 02314665, 02314657, 02386844, 02386887, 02386879, 02386895, 02386852, 02275813, 02275821, 02275848, 02275856, 01937413, 02280345, 01937383, 01937391, 1937405, 02396718, 02396742, 02396726, 02396696, 02396734, 02341387, 02341379, 0341395, 02341417, 02341409, 02330105, 02330113, 02330156, 02330148, 023301210, 2327120, 023271550, 02327163, 02327147, 02327163, 002327112, 02282941, 02282968, 02282984, 02311925, 02282976<br>Hydromorphone:<br>02359502, 02359510, 02125323, 02125331, 02125366, 02243562, 00627100, 00622133, 02145901, 02145928, 00786535, 01916386, 02364115, 02364158, 02364131, 02364123, 00705438, 00786543, 00125121, 00125083, 00885444, 00885428, 00885401, 00885436, 02319438, 02319411, 02313403<br>Morphine:<br>00632503, 00690198, 00690201, 02019957, 02019949, 02177749, 02019930, 02019965, 00392588, 0392561, 02242484, 00850330, 00617288, 00591467, 00591475, 00621935, 02184443, 021884435, 02242163, 02184451, 02014300, 02014327, 02014319, 02014297, 02015439, 02244791, 02244790, 0244792, 02302780, 02302764, 02302799, 02302799, 02302772, 02302802, 02014238, 02014254, 00594652, 00594644, 00594636, 00675962<br>Oxycodone:<br>02231934, 02240132, 02240131, 02319977, 02319993, 02319985, 00443948, 02262983, 00789739, 02324628, 01916548, 01916475, 00608165, 02307898, 01916572, 00608157<br>Tramadol:<br>02450429, 02450437, 02450445, 02296381, 02296403, 02296411, 02426153, 02349469, 02336790, 02439050, 02383209, 02388308, 02388324, 02389800, 02401657, 02347180, 02264846
